# *Mycobacterium haemophilum *osteomyelitis: case report and review of the literature

**DOI:** 10.1186/1471-2334-6-70

**Published:** 2006-04-10

**Authors:** Sameer Elsayed, Ron Read

**Affiliations:** 1 9-3535 Research Rd NW, Calgary, Alberta, Canada T2L 2K8

## Abstract

**Background:**

*Mycobacterium haemophilum *is a slow-growing, fastidious, iron-requiring microorganism that, relative to other non-tuberculous mycobacterial species, has rarely been documented as a cause of human infection. This microorganism appears to be acquired via environmental exposure although its natural habitat and mode of acquisition are unknown. It has primarily been implicated as a cause of ulcerating cutaneous or subcutaneous nodular skin lesions, particularly in immunocompromised patients, although infections at extracutaneous sites have also been described. Osteomyelitis, while rarely documented, appears to be an important complication of infection with *M. haemophilum *in these patients.

**Case presentation:**

We describe a unique case of culture-confirmed *M. haemophilum *osteomyelitis in an adult woman with polycythemia vera and review the world literature on bone infections due to this organism.

**Conclusion:**

*Mycobacterium haemophilum *is an important but infrequently encountered cause of osteomyelitis in immunocompromised patients, often requiring months to years of medical therapy, with or without surgery, to effect a clinical cure.

## Background

*Mycobacterium haemophilum *is a slow-growing, fastidious, iron-requiring microorganism that, relative to other non-tuberculous mycobacterial species, has rarely been documented as a cause of human infection. This microorganism appears to be acquired from environmental exposure although its natural habitat and mode of acquisition are unknown. It has primarily been implicated as a cause of ulcerating cutaneous or subcutaneous nodular skin lesions, particularly in immunocompromised patients, although infections at extracutaneous sites have also been described. Like other nontuberculous mycobacteria, *M. haemophilum *can cause chronic cervicofacial lymphadenitis, particularly in immunocompetent children [1-3]. Osteomyelitis, while rarely documented, appears to be an important complication of infection with *M. haemophilum *in these patients. We report a case of *M. haemophilum *osteomyelitis in a patient with polycythemia vera and provide a summary review of the world literature on *M. haemophilum *bone infections.

## Case presentation

A 56-year-old businesswoman with a 16-year history of polycythemia vera, apparently well controlled with busulfan, presented for medical attention with a 1-month history of painful ulcerating nodular skin lesions on her right wrist and right ankle. The lesions were biopsied, with Ziehl-Neelsen stains demonstrating the presence of acid-fast bacilli (AFB). She had lived in Canada for most of her adult life aside from yearly, extended vacations in Arizona, USA. She was not aware of any contact with tuberculous individuals, and had no history of exposure to fish tanks. She recalled experiencing minor trauma to her right wrist just prior to the emergence of the nodular lesions. A Mantoux test was not performed. Physical examination revealed tender nodular and erythematous ankle and wrist lesions (3 cm and 1 cm in diameter, respectively), with full thickness skin ulceration. Chest X-ray was normal. While awaiting culture results, she was started on clarithromycin 500 mg orally twice daily, ciprofloxacin 500 mg orally twice daily, and rifabutin 300 mg orally once daily, after which her wrist lesion slowly healed. The ankle lesion did not respond significantly to medical therapy. X-rays demonstrated osteolysis of the distal tibia consistent with osteomyelitis. She developed gastrointestinal intolerance to ciprofloxacin and rifabutin, and was continued on clarithromycin alone. At this time, her polycythemia treatment was subsequently switched from busulfan to hydroxyurea. The wrist and ankle bone biopsy specimens were inoculated onto plain and hemin-supplemented Middlebrook 7H10 agar media and BACTEC 12B blood culture bottles incubated at 30°C in an aerobic atmosphere (National Mycobacteriology Laboratory, Edmonton, Canada). After 1 and 6 weeks, respectively, growth of acid-fast bacilli was observed only on the hemin-supplemented Middlebrook slants inoculated with the wrist and ankle specimens and definitively identified as *M. haemophilum *by high-performance liquid chromatography (Laboratoire de sante publique du Quebec, Montreal, Canada). Susceptibility testing was performed using Etest (AB BIODISK, Solna, Sweden) on Mueller-Hinton agar supplemented with sheep blood. The organism was susceptible to clarithromycin (MIC ≤ 16 μg/ml) and rifabutin (MIC ≤ 0.12 μg/ml) but resistant to ciprofloxacin (MIC 4 μg/ml), amikacin (MIC ≥ 8 μg/ml), and ethambutol (MIC ≥ 8 μg/ml) based on unofficial breakpoints proposed by the National Mycobacteriology Laboratory, Edmonton, Canada. Re-biopsy of the ankle lesion demonstrated the presence of AFB but no evidence of superinfection with other pathogens. An HIV test was ordered at this time but was negative. An MRI scan 6 months later demonstrated a significant soft tissue inflammatory mass underlying the ulcer with extension through the cortex of the tibia and into the marrow cavity. After debridement, the ulcer began to granulate and heal over. After one year of follow-up, our patient's ankle lesion had completely epithelialized, aside from the development of a small intermittently draining sinus. Repeat MRI another 6 months later demonstrated resolution of the original soft tissue inflammatory mass but without evidence of bony healing. She improved after 2 years of clarithromycin therapy, with plans to continue therapy indefinitely until there was radiologic evidence of bony healing, although she eventually died of transformation to acute leukemia.

## Discussion

*Mycobacterium haemophilum *is a slow-growing, fastidious, nontuberculous mycobacterial species that was first isolated and described by Sompolinsky in 1978, who recovered the organism from chronic ulcerating subcutaneous lesions in a woman with Hodgkin's disease [4]. Since then, approximately 100 cases of infection have been described worldwide, with the majority of affected individuals being immunocompromised by virtue of organ or bone marrow transplantation, haematological malignancy, or advanced HIV infection/AIDS [2,5-12]. In such individuals, the classical clinical presentation has been that of multiple tender ulcerating cutaneous or subcutaneous nodular skin lesions, commonly on the extremities and often overlying joints [2,5-12]. Occasionally, lesions have been associated with cellulitis or complicated by abscess formation, fistula development, osteomyelitis, septic arthritis, or bacteremia [2,5-7,9-11]. Cases of pneumonia, pulmonary nodules, or sinusitis without skin lesions have also been described in both immunocompromised and immunocompetent individuals [2,3,5-7,9-12]. Several cases of localized lymphadenitis, particularly of the cervical, submandibular, and perihilar regions, have been reported in immunocompetent patients, especially children [1-3].

The natural habitat and mode of acquisition of *M. haemophilum *are unknown. However, the geographic distribution of *M. haemophilum *is thought to be ubiquitous [2,9]. Evidence from reported cases points to an environmental reservoir, possibly aquatic, although attempts to recover the organism through environmental sampling have been unsuccessful [2,13]. A few patients have reported antecedent trauma at the site of infection [11,13]. Although the pathophysiology of *M. haemophilum *infection is not well understood, cell-mediated immunity appears to play a key role in disease pathogenesis and outcome [10,11].

In common with most other nontuberculous mycobacterial infections, disease is usually chronic [2]. The organism is difficult to cultivate in the laboratory, and is unique among the mycobacteria in its requirement for iron-containing compounds in growth media [2]. Furthermore, *M. haemophilum *requires low incubation temperatures (30–32°C) for growth [2]. Hence, cases of infection due to *M. haemophilum *are likely under-reported. However, advances in molecular biology have greatly facilitated our understanding of the clinical spectrum of infection caused by this microorganism and have paved the way for sensitive and specific detection of *M. haemophilum *directly from clinical specimens or for definitive characterization of suspect clinical isolates using tools such as PCR, real-time PCR, or 16S ribosomal RNA gene sequencing [1,14,15].

*Mycobacterium haemophilum *appears to be the most important cause of non-tuberculous mycobacterial osteomyelitis infections in humans [6,16-26]. Over 30 cases of *M. haemophilum *bone infections have been described to date, with most occurring in patients with advanced HIV disease or bone marrow/solid organ transplants (Table 1) [2,5-7,9-12,17-28]. Infections may involve multiple sites, are frequently associated with septic arthritis and/or overlying cutaneous infection, and usually involve the bones of the foot, ankle, knee, elbow, and fingers [2,5-7,9-12,17-28]. These and other sites may be involved via contiguous spread or hematogenous dissemination from a pulmonary or cutaneous source. Affected patients may or may not report a history of antecedent trauma. Infections of bone typically develop over the course of several weeks. Plain film radiographs and MRI scans often reveal well-marginated osteolysis, cortical bone destruction, and adjacent soft tissue inflammation [21]. In a 3-year review of atypical mycobacterial skeletal infections in 25 HIV-infected patients, *M. haemophilum *accounted for 44% (11/25) of cases, followed by infection with *M. kansasi *and *M. avium-intracellulare *[6]. A study from January 1989 to September 1991 at seven metropolitan hospitals in New York City identified 13 patients with culture-confirmed *M. haemophilum *infections (11 with HIV infection and 2 with bone marrow transplants), of which 6 had osteomyelitis [11]. While our case may be the first report of *M. haemophilum *osteomyelitis in a patient with polycythemia vera, our patient's predisposition to infection was likely a result of myelosuppression from long-term busulfan/hydroxyurea therapy. A case of fatal disseminated non-*M. haemophilum *atypical mycobacterial infection was reported in a woman with pulmonary fibrosis secondary to long-term busulfan use [29].

**Table 1:** Summary of reported cases of *M. haemophilum *osteomyelitis in the world literature.

Country (Year)	Age/Sex	Underlying Disease(s)	Anatomic site	Therapy (duration)	Outcome	Reference(s)
Australia (1979)	58/M	Lymphoma	foot	INH, RIF, ETH (N/A)	Partial response	[22]
Australia (1979)	55/F	Renal transplant	ankle	INH, RIF, ETH (N/A)	Died	[22]
Australia (1990)	39/M	AIDS, Hodgkin's disease	foot	AK, CIP, DOX, RIF (10 weeks)	Cure	[6, 25]
Brazil (2000)	30/M	AIDS	elbow	N/A	N/A	[9]
France (1982)	48/M	Renal transplant	finger	Surgery; MIN, ERY (> 2 months)	Cure	[27]
France (1984)	48/M	Renal transplant	ankle	RIF, MIN (N/A)	Cure	[2, 7, 27]
France (1992)	N/A	AIDS	fingers, toes, tibia, elbow, thoracic vertebrae	INH, RIF, ETH (N/A)	No improve-ment	[6, 32]
Germany (2002)	53/F	AIDS	tibia	ETH, RIF, CLR	Cure	[33]
USA (1983)	N/A	Cardiac transplant	wrist, lower extremity	No treatment	Death from chronic graft rejection	[2, 5, 17]
USA (1984)	30/F	Renal transplant AIDS	hand	INH, RIF, ETH, MIN (N/A)	Died	[2]
USA (1990)	37M	AIDS	ankle	RIF, ETH. CIP, AK, DOX (14 months)	Cure	[6, 7, 10, 26, 28]
USA (1990)	38/M	AIDS	N/A	AK, CIP, EMB, RIF (N/A)	Resolved	[7, 11]
USA (1990)	49/M	AIDS	tibia	INH, RIF, ETH, CIP, CLO, AK (N/A)	Died	[6]
USA (1990)	77/M	T-cell lymphoma	hand	Surgery (curettage only)	Relapse; Died from lymphoma complica-tions	[2]
USA (1991)	32/F	AIDS	N/A	CLR, MIN, RIF (N/A)	Improved; responded then relapsed	[7, 11]
USA (1991)	32/F	AIDS	arm	CIP, INH, RIF, PZA (N/A)	Resolution	[11]
USA (1991)	30/M	AIDS	N/A	AK, CLR, CLO, ETH, INH, RIF (N/A)	Responded then relapsed	[7, 11]
USA (1991)	51/M	AIDS	N/A	CIP, DOX, ETH, INH, PZA, RIF (N/A)	Resolved	[7, 11]
USA (1991)	37/M	AIDS	N/A	AK, DOX (N/A)	Died	[11]
USA (1992)	44/M	AIDS	tibia; fibula	AK, CIP, CLO, ETH, INH, RIF (N/A)	Responded then relapsed	[6, 7, 18]
USA (1992)	30/F	AIDS	3^rd ^finger; calcaneous	RIF, CIP, DOX, AK (17 months)	Cure	[6, 26]
USA (1992)	21/F	AIDS	tibia	MIN, RIF (N/A)	Responded	[2, 6, 19]
USA (1993)	29/F	AML, BMT	N/A	CIP, CLR, RB (6 months)	Cure	[2, 10, 12]
USA (1993)	33/M	AIDS	lower extremity	ETH, CLR, CIP, AK (N/A)	Died of AIDS complica-tions	[2]
USA (1994)	35/M	AIDS	N/A	CIP, CLR, RYF (5 months)	Unknown	[10]
USA (1994)	41/M	AIDS	olecranon	RIF, INH, PYZ (N/A)	Resolved in 9 months	[6, 24]
USA (1996)	46/M	AIDS	foot	CIP, RB, CYC, AZI	Improved after treatment	[20]
USA (1997)	20/M	Cardiac transplant	olecranon	CLR, RIF (N/A)	Cure	[5, 23]
USA (1998)	36/M	AIDS	femur	N/A	N/A	[21]
USA (2000)	47/F	AA, BMT	N/A	CIP, CLR, DOX, RYF (> 6 months)	Improved	[10]
USA (1993)	33/M	AIDS	lower extremity	ETH, CLR, CIP, AK (N/A)	Died of AIDS complica-tions	[2]

AA, Aplastic anemia; AIDS, acquired immunodeficiency syndrome; AK, amikacin; AML, acute myelocytic leukemia; AZI, azithromycin; BMT, bone marrow transplant; CIP, ciprofloxacin; CLO, clofazamine; CLR, clarithromycin; CYC, cycloserine; DOX, doxycycline; ETH, ethambutol; INH, isoniazid; MIN, minocycline; PYZ, pyrazinamide; RB, rifabutin; RIF, rifampin; RYF, rifamycin (rifampin or rifabutin). N/A = information not available.

Most strains of *M. haemophilum *demonstrate in-vitro susceptibility to ciprofloxacin, clarithromycin, rifamycins, and clofazimine [2,7,8,12,30]. Isolates are usually resistant to isoniazid, ethambutol, and pyrazinamide, while susceptibility to doxycycline, minocycline, amikacin, and para-aminosalcylic acid is variable [2,7,8,12,30]. There is no consensus on the optimal modality or duration of treatment of *M. haemophilum *bone infections. However, successful treatment of osteomyelitis may require several months of antimicrobial therapy but should be guided by the patient's underlying condition and clinical response. Based on information from previously reported cases, combinations of drugs, including ciprofloxacin, clarithromycin, and/or rifabutin appear to be associated with the greatest potential success when used for the treatment of osteomyelitis or localized skin and soft tissue infection [2,5-7,10-12,20,31]. Patients should be monitored periodically while on combinations of rifamycins and macrolides due to the potential for drug-drug interactions. Rifabutin induces hepatic cytochrome P-450 enzymes, which may result in increased metabolism of clarithromycin, leading to potentially subtherapeutic concentrations of the latter drug. However, clarithromycin inhibits the hepatic cytochrome P-450 enzymatic pathway, leading to potentially toxic concentrations of rifabutin [23,31]. Rifamycins should not be used alone due to the potential for rapid development of resistance. Patients with localized disease usually respond favorably to medical treatment, although deaths have been reported, especially for disseminated infection [2,7,8,10,12]. It has been observed that improvement of immune function during the course of disease may lead to an improve outcome [9] but this has not been well documented in osteomyelitis cases. Surgery has occasionally been used, often in combination with antimicrobial agents, for the treatment of *M. haemophilum *bone infections but the impact of surgery on patient outcome is unknown.

## Conclusion

*M. haemophilum *is a clinically significant mycobacterial species with a predilection for causing bone infection in immunocompromised individuals. In the appropriate clinical setting, *M. haemophilum *osteomyelitis should be considered in the differential diagnosis of immunocompromised patient presenting with nodular or ulcerative skin lesions in conjunction radiographic evidence of adjacent bone destruction, especially if tissue biopsies reveal the presence of AFB. Successful outcomes usually require months or even years of medical therapy.

## Competing interests

The author(s) declare that they have no competing interests.

## Authors' contributions

RR was directly involved in the patient's care. SE was the laboratory physician involved in the patient's care. SE performed the literature review. Both authors wrote the manuscript.

## Pre-publication history

The pre-publication history for this paper can be accessed here:


